# Supplementary material for: Differentiating attack-defense performance for starting and bench players during the Tokyo Olympics men’s basketball competition
Source: PLoS One. 2023 Dec 15;18(12):e0296012. doi: 10.1371/journal.pone.0296012 (PMC10723665; doi:10.1371/journal.pone.0296012)
Supplement: Attachment — Submitted filename: Response to Reviewers.docx [file pone.0296012.s002.docx]

**Response to Reviewers**

Thank you for inviting us to resubmit a revised draft of our manuscript entitled, "Differentiating attack-defense performance for starting and bench players during the Tokyo Olympics men's basketball competition" to PLOS ONE. We also appreciate the time and effort you and each of the reviewers have dedicated to providing insightful feedback on ways to strengthen our paper. We have incorporated changes that reflect the detailed suggestions you have graciously provided. We also hope that our edits and the responses we provide below satisfactorily address all the issues and concerns the reviewers have noted.

To facilitate your review of our revisions, the following is a point-by-point response to the questions and comments delivered in your letter.

**Review Comments to the Author**

Reviewer #2: I thank the authors for their efforts to respond to each of my comments, you have done a very good job. I have just some minor comments for the last version of the manuscript.

**Response:** We greatly appreciate the time and effort you put into our manuscript, as well as your valuable comments.

There are some minor English language issues in the manuscript. Please revise it.

**Response:** Thank you for this suggestion. Some English language issues have been corrected in our revised manuscript (see line 32-33,41,57,60,64,69,89,101-104,113,123,147,174,201,213,246,262,316,326, and 334).

In the statistical analysis section. Please state the analysis for testing the normality of the variable’s distribution. Also, you need to acknowledge which test you have used to analyze the homogeneity of variance for the independent t-tests.

**Response:** Thank you for this suggestion. We used Shapiro-Wilk test to test the normality distribution of the dependent variables in this study. Also, Levene’s test was used to test the homogeneity of variance. We have included these in the revised manuscript (see line 127-129).

Finally, unless otherwise, you only need to mention the alpha level once (there is no need to mention the significance level for each test).

**Response:** Thank you for this suggestion. Based on your suggestion, we have corrected the relevant paragraph in the revised manuscript (see line 129-130), and addressed the issue you mentioned above.

L132-134: This paragraph is a bit odd. Could you rephrase it?

**Response:** Thank you for this suggestion. We have re-written this paragraph in our revised manuscript (see line 135-137).

In the results section, you describe again the comparisons and correlations procedures (e.g., L168, L176, and L198). In the results section, the reader is expecting the results of your analysis and not the description of it. Thus, I would suggest describing comprehensively your statistical analysis and comparisons in the proper section to avoid repeating information.

**Response:** Thank you for this suggestion. It is really true as you mentioned that there is some repeating information in the results section. We have deleted the contents of lines 168, 176, 198 and 213, and also corrected some of the expressions in the revised manuscript (see line 172-173,187,200-201,213-214). Additionally, considering your suggestion, we have detailed the statistical analysis of this study in Data analysis section (see line 124-137), and give the reader a clearer understanding of how each statistical method was applied in this study.

Special thanks to you for your good comments and suggestions.

We appreciate for Editors/Reviewers’ warm work earnestly, and hope that this correction will meet with approval.

Once again, thank you for giving us the opportunity to strengthen our manuscript with your valuable comments and queries.

Sincerely,

Sun wenping

Universiti Putra Malaysia

Department of Sport Studies, Faculty of Educational Studies

Kuala Lumpur, Malaysia

[suncao850525@gmail.com](mailto:suncao850525@gmail.com)

086-15236066180
